# The phospholipase DDHD1 as a new target in colorectal cancer therapy

**DOI:** 10.1186/s13046-018-0753-z

**Published:** 2018-04-13

**Authors:** Stefania Raimondo, Marta Cristaldi, Simona Fontana, Laura Saieva, Francesca Monteleone, Giovanna Calabrese, Gianluca Giavaresi, Rosalba Parenti, Riccardo Alessandro

**Affiliations:** 1 Dipartimento di Biopatologia e Biotecnologie Mediche, Sezione di Biologia e Genetica, University of Palermo, Palermo, Italy; 2 Department of Surgical, Oncological and Stomatological Disciplines, Sector of Clinical Research in Oral Medicine, University of Palermo, Palermo, Italy; 3 Department of Biomedical and Biotechnological Sciences, Physiology Section, University of Catania, Catania, Italy; 4 IRCCS Rizzoli Orthopedic Institute, Innovative Technologic Platform for Tissue Engineering, Theranostics and Oncology, Palermo, Italy; 5 Institute of Biomedicine and Molecular Immunology (IBIM), National Research Council, Palermo, Italy

**Keywords:** *Citrus*-limon nanovesicles, Phospholipase DDHD1, Colorectal cancer

## Abstract

**Background:**

Our previous study demonstrates that *Citrus*-limon derived nanovesicles are able to decrease colon cancer cell viability, and that this effect is associated with the downregulation of the intracellular phospholipase DDHD domain-containing protein 1 (DDHD1). While few studies are currently available on the contribution of DDHD1 in neurological disorders, there is no information on its role in cancer. This study investigates the role of DDHD1 in colon cancer.

**Methods:**

DDHD1 siRNAs and an overexpression vector were transfected into colorectal cancer and normal cells to downregulate or upregulate DDHD1 expression. In vitro and in vivo assays were performed to investigate the functional role of DDHD1 in colorectal cancer cell growth. Quantitative proteomics using SWATH-MS was performed to determinate the molecular effects induced by *DDHD1* silencing in colorectal cancer cells.

**Results:**

The results indicate that DDHD1 supports colon cancer cell proliferation and survival, since its downregulation reduces in vitro colon cancer cell viability and increases apoptosis rate, without affecting normal cells. On the contrary, in vivo studies demonstrate that the xenograft tumors, derived from DDHD1-overexpressing cells, have a higher proliferation rate compared to control animals. Additionally, we found that functional categories, significantly affected by DDHD1 silencing, were specifically related to cancer phenotype and for the first time associated to DDHD1 activity.

**Conclusions:**

In conclusion, this study provides the first evidence confirming the role of DDHD1 in cancer, providing a possibility to define a new target to design more effective therapies for colon cancer patients.

**Electronic supplementary material:**

The online version of this article (10.1186/s13046-018-0753-z) contains supplementary material, which is available to authorized users.

## Background

It is widely known that cancer is a multifactorial disease able to affect multiple aspects of cellular metabolism. Abnormalities in lipid and glucose metabolism are frequently shared by tumors, even if different in types and origin [1], and often represent target for developing anti-cancer therapies. Our recent study focuses on the anti-cancer effects of *Citrus*-limon derived nanovesicles on colon cancer cells [2] and, after a proteomic analysis, we found a decrease of lipid metabolism in treated cells. Interestingly, the anti-cancer effects observed after nanovesicle treatment correlate to the downregulation of the phospholipase DDHD1 [3].

The phospholipase A1 (PLA1) family is implicated in different intracellular mechanisms and is classified as intracellular and extracellular proteins. The intracellular DDHD1 was identified as a phosphatidic acid (PA)-preferring PLA1 (PA-PLA1) [4] involved in the synthesis of lysophospholipid mediators. More precisely, Yamashita et al. demonstrated that DDHD1 activity is augmented by phospholipase D, which is responsible for the increase of PA levels [5]. In turn, PA is able to induce DDHD1 activity, which catalyzes the generation of lysophosphatidylinositols (LPIs) by hydrolysis of phosphatidylinositol (PI) at acyl group on ester positions sn-1 [5]. Studies have proven that LPI, produced from DDHD1 hydrolysis of PI, is involved in different processes, and significant data has been collected on its role in tumorigenesis. Based on the first evidence of their implication in cancer, obtained in 1994 from Falasca’s research team [6], several in vitro and in vivo studies, as well as clinical evidence, demonstrated the central role of LPIs in neoplastic transformation of different types of cancer such as ovarian, peritoneal and prostate cancer. Specifically, LPI activity has shown to be mediated by the interaction with the G protein-coupled receptor 55 (GPR-55), a cannabinoid receptor also described as correlated to tumor growth and aggressiveness [7–10].

Although a possible involvement of DDHD1 in the LPI-GRR55 axis has been previously postulated [11], information about the involvement of the phospholipase in cancer is missing.

In this study we provide the first evidence of the involvement DDHD1 in cancer, elucidating its role in supporting tumor cell proliferation and survival. We demonstrated that silencing of *DDHD1* by small interference RNA reduces in vitro colon cancer cell viability and increases apoptotic cell death through the inhibition of MAPK/ERK and PI3K/Akt signaling pathways. Additionally, DDHD1 overexpression supports in vitro and in vivo cancer cell growth. Finally, a proteomic analysis of silenced cells opens up to the opportunity to investigate and possibly define the molecular effects of *DDHD1* silencing.

In conclusion, for the first time our results show that DDHD1 is responsible for colon cancer cell growth, even though future studies will be needed in order to better understand and clarify the mechanism by which it acts on neoplastic transformation.

## Methods

### Cell culture and reagents

The human colorectal adenocarcinoma cell lines, SW480 and HCT-116, and the human bone marrow-derived stromal cell line, HS5, were obtained from ATCC (Manassas, VA, USA). SW480 and HCT-116 cell lines were cultured in RPMI 1640 medium (Euroclone, UK), HS5 cell line was cultured in DMEM (Euroclone, UK), supplemented with 10% fetal bovine serum (Euroclone, UK), 2 mM L-glutamine, 100 U/ml penicillin and 100 mg/ml streptomycin (Euroclone, UK). Human Umbilical Vein Endothelial Cells (HUVEC) were obtained from Lonza and grown in Endothelial Growth Medium (EGM, Clonetics, Verviers, Belgium) according to supplier’s information.

### SiRNA cell transfection

SW480, HCT116, HS5 and HUVEC cells were transiently transfected with 10 nM of scrambled siRNA (negative control) or *DDHD1* siRNA (Dharmacon RNA Technologies, Lafayette, CO). Lipofectamine RNAiMAX Transfection Reagent was used for siRNA transfection according to manufacturer’s indications (Thermo Fisher Scientific).

### Bio-plex Pro Magnetic Cell Signaling Assay

Levels of ERK 1/2, phospho-ERK 1/2, Akt and phospho-Akt were determined in the cell lysate of SW480 cells transfected with scrambled or DDHD1 siRNA using the Bio-Plex Pro Cell Signaling Assay (Bio-Rad, Hercules, CA), according to the manufacturer’s instructions. Measurement are provided as the median fluorescence intensity (MFI) for a given bead population. Assay was developed using the Bio-plex 200 system and the data acquisition was done using Bioplex Manager Software.

### Flow cytometry

Phosphorylation levels of Akt in SW480 cells transfected with scrambled or DDHD1 siRNA were determined by flow cytometry. Cells were fixed and permeabilized with Leucoperm kit (AbDSerotec). Akt- and phospho-Akt unconjugated primary antibody (Santa Cruz Biotechnology, Santa Cruz, CA, USA) was added; cells were washed and a FITC secondary antibody was added. Stained cells were analyzed on a FACS Calibur (Becton Dickinson) using Cellquest software.

### Proteomic analyses: sample preparation, SWATH-MS and data analysis

SW480 cells transiently transfected with scrambled or *DDHD1* siRNA, were dissolved in 100 μL of 50% tetrafluoroethylene (Sigma-Aldrich) in PBS and subjected to tryptic digestion. Three biological replicates of each sample were prepared and subjected to DDA and SWATH analysis. A deep description of the tryptic digestion and DDA/SWATH procedures are reported in Additional file 1: Supplementary Material and Methods. DDA raw files were combined and searched against the human database to generate the reference spectral library, which was used for SWATH data processing and quantification. The protein list with FDR lower than 5% generated by analyzing SWATH data with PeakView 2.2, was exported to MarkerView 1.2.1 for statistical data analysis using a pairwise t-test. Fold Change (FC) Ctrl-SW480 vs shDDHD1-SW480 thresholds at 1.5 with *p*-value ≤ 0.05 were used to consider a protein up- or down-regulated. GraphPad Prism 7.00 for Windows to make a volcano plot scaling in which the FC was transformed using the log2 function, so that the data is centered on zero, while the *p*-values was −log10 transformed.

The Gene Ontology and KEGG pathway analysis of proteins down-regulated by *DDHD1* silencing was performed using the online tool DAVID (http://david.abcc.ncifcrf.gov/) [12] and the ClueGO v2.3.3 + CluePedia v1.3.3, a Cytoscape v3.4.0 plug-in was used to visualize GO terms and pathways in functionally organized networks reflecting the relations between the biological terms based on the similarity of their linked gene/proteins [13, 14]. Details of the bioinformatics analysis are described in Additional file 1: Supplementary Material and Methods.

For the enrichment of biological terms and groups, we used the two-sided (Enrichment/Depletion) tests based on the hyper-geometric distribution. We set the statistical significance to 0.05 (*p* ≤ 0.05), and we used the Benjamini-Hochberg adjustment to correct the *p*-value for the terms and the groups created by ClueGO. The parameters used were: kappa score threshold set to 0.4; GO tree interval: 3–8; Leading Group: Highest Significance.

### Plasmid transfection protocol

PCMV6 plasmid, over-expressing DDHD1 sequence, was purchased from TrueORF Gold, OriGene and used to transform DH5alpha cells (*Escherichia coli*). Plasmids were isolated using EuroGold Plasmid MiniPrep Kit (Euroclone, UK). SW480 cells were seeded in 6-well plates; after 24 h, cell transfection was performed with 2,5 μg of mock (negative control) or *DDHD1* plasmid DNA according to Lipofectamine 3000 protocol (Life Technologies, California, US). Transfection efficiency was evaluated by transfection with pINCO plasmid expressing Green Fluorescent Protein (GFP). About 90% of transfection efficiency was obtained. The cells were expanded and then selected with 750 μg/ml of neomycin for 2 weeks.

### Human colon cancer mouse xenograft

Five weeks old female Athymic Nude-Foxn1nu mice (*n* = 10) were purchased from Harlan (Harlan Laboratories, San Pietro al Natisone, Italy) and acclimated for a week prior to experimentation. Each mouse was inoculated subcutaneously in the right flank with SW480 transfected with mock or DDHD1 plasmid DNA (3 × 10^6^) suspended 1:1 in a solution containing PBS and Matrigel (BD Biosciences) in a final volume of 0.2 ml. Xenograft tumors were measured and mice were weighed twice a week, for 2 weeks. Tumor volume was determined by caliper by using the following formula: L X W^2^/2 = mm^3^ where L and W are the longest and shortest perpendicular measurements in millimeters, respectively. The animals were euthanized at day 15 and the tumor resected.

### Statistical analysis

Data are expressed as means ± SD of three independent experiments. Statistical analysis was done with a paired sample t-test. Differences were considered significant when *p* ≤ 0.05.

Regarding the analysis of volume tumor data, after having evaluated the normal distribution (Shapiro-Wilk test) and homogeneity of variance (Levene test), volumetric data were analyzed with a generalized linear model (GLM) for repeated measures by considering the between-subject factor constituting the design of the adopted experimental model: ‘treatment’ - two levels (mock, over DDHD1); and the within-subject factor ‘experimental time’ – four levels (starting point, 8, 12 and 15 days). The best model was obtained, verifying both the main effects of between and within-subject factors, and their interactions. Pairwise comparison tests were carried out by using adjusted Sidak test.

## Results

### DDHD1 silencing reduces in vitro colon cancer cell viability and increases apoptotic cell death

Our recent study demonstrates that the anticancer effects induced by lemon nanovesicles on colon cancer cells are associated with a downregulation of the phospholipase DDHD1. In order to investigate the role of DDHD1 in colon cancer, the first approach was to use a small interference RNA. We transiently transfected SW480 and HCT-116 cancer cell lines, as well as non-tumor cell lines HS5 and HUVEC, with *scrambled* (negative control) or *DDHD1* siRNA and analyzed them after 48 and 72 h. As shown in Additional file 2: Figure S1, both *DDHD1* mRNA and protein levels decreased in cancer as well as in non-tumor cells after siRNA transfection.

**Fig. 1:**
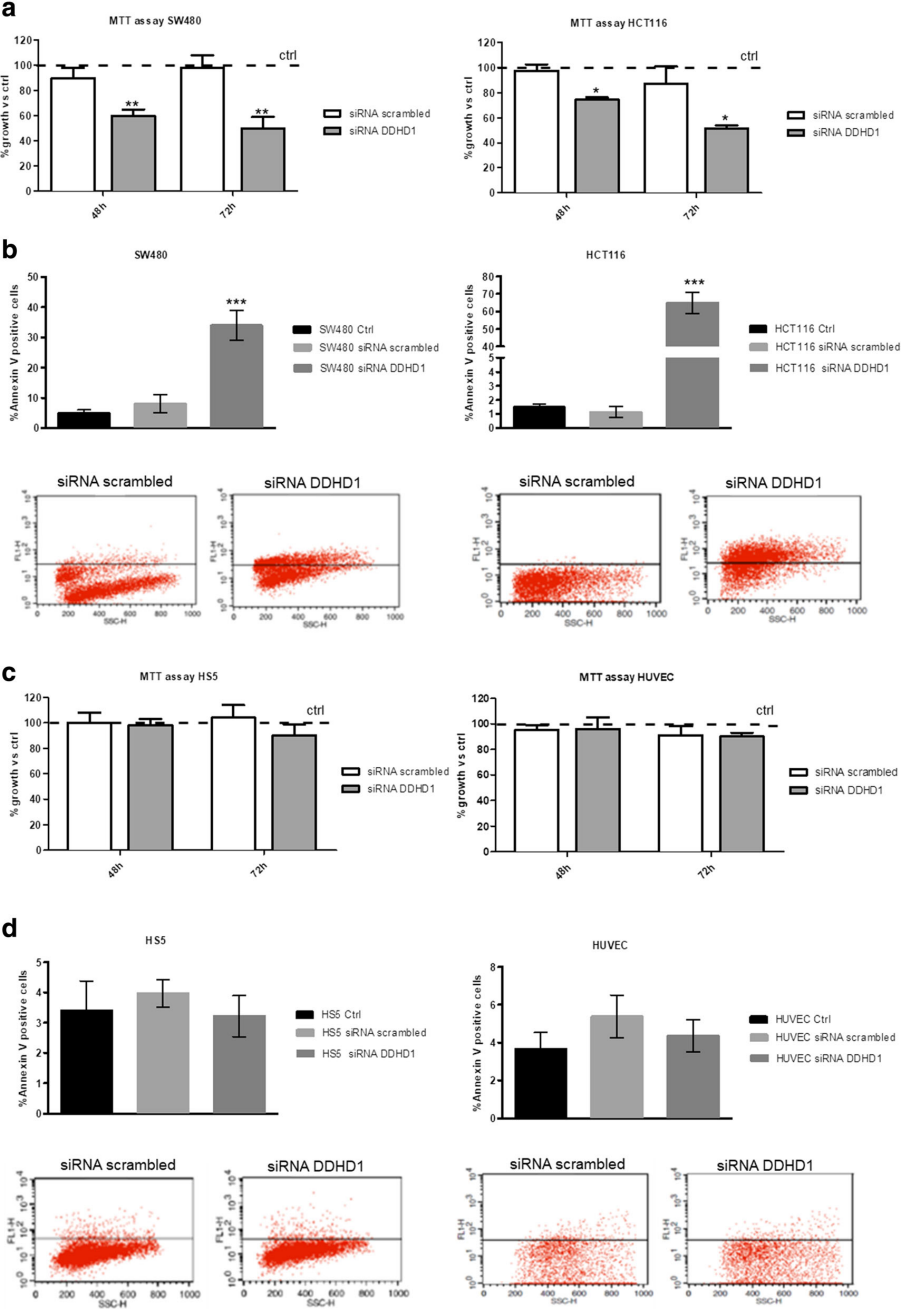
Effects of DDHD1 silencing on tumor and non-tumor cell viability and apoptosis. **a**, **c** Cell viability was measured by MTT assay on SW480, HCT116, HS5 and HUVEC cell lines after 48 or 72 h of transfection with scrambled siRNA or with *DDHD1* siRNA. The values were plotted as % of growth vs Ctrl (non transfected cells). Each point represents the mean ± SD of three independent experiments. **b**, **d** Cell death was detected by Annexin V staining after 72 h of transfection with scrambled siRNA or with DDHD1 siRNA. Histogram reported % of Annexin V positive cells in transfected samples compared to untreated (Ctrl). Asterisks indicate statistically significant values in comparison to cells transfected with scrambled siRNA (*p ≤ 0.05; **p ≤ 0.01; ***p ≤ 0.001)

To test the in vitro effects of *DDHD1* silencing on cancer cell growth and cell death, we evaluated cell viability and apoptosis rate respectively using MTT and Annexin V assay (Fig. 1a and b). Forty-eight hours after transfection, both SW480 and HCT-116 cancer cells showed a decreased level of cell viability (around 40%); the effects increased after 72 h of siRNA-DDHD1 transfection (more than 50%) (Fig. 1a). Also, the rate of cell apoptosis was increased after transfection (35% Annexin V positive cells in SW480, 60% in HCT116) (Fig. 1b). Interestingly, cell growth and apoptosis rate in normal cells, HS5 and HUVEC, were not affected by *DDHD1* silencing (Fig. 1c and d). This data suggests that the effect of *DDHD1* silencing is tumor specific.

### DDHD1 silencing inhibits MAPK/ERK and PI3K/Akt signaling

**Fig. 2:**
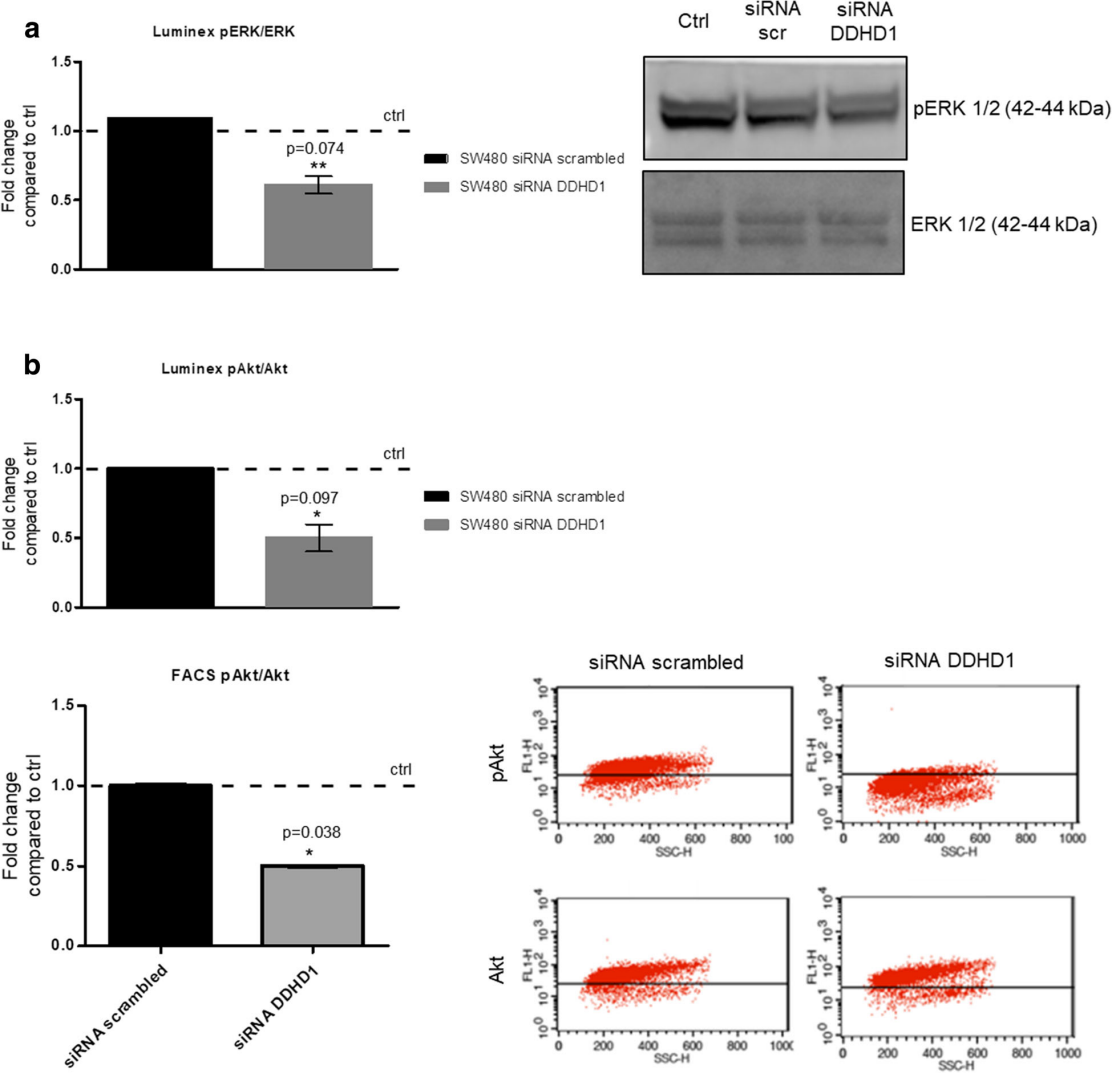
Effects of DDHD1 silencing on ERK and Akt signaling pathways. **a** Levels of ERK 1/2, phospho-ERK 1/2 were determined in the cell lysate of SW480 transfected with scrambled or DDHD1 siRNA using the Bio-Plex Pro Cell Signaling Assay (left panel) and western blot (right panel). **b** Levels of Akt and phospho-Akt were determined by the Bio-Plex Multiple Cytokine Assay (upper panel) and by FACS analysis (lower panel) in the SW480 transfected with scrambled or DDHD1 siRNA (*p ≤ 0.05; **p ≤ 0.01)

To investigate if the reduction of cell viability was correlated to inhibition of canonical proliferation and survival pathways, we evaluated the phosphorylation rate of ERK and Akt, using the Bio-plex Pro Magnetic Cell Signaling Assay. The levels of ERK 1/2, phospho-ERK 1/2, Akt and phospho-Akt were measured in SW480 cell lysates, previously transfected with scrambled or *DDHD1* siRNA. A decrease of pERK/ERK (0.5-fold decrease, Fig. 2a, left panel) as well as pAkt/Akt (0.5-fold decrease, Fig. 2b, upper panel) ratio was observed in cells after *DDHD1* silencing. Next, ERK and Akt phosphorylation levels were measured respectively using Western blotting (Fig. 2a, right panel) and FACS analysis (Fig. 2b, lower panel), confirming the results obtained through the Bio-plex Pro Magnetic Cell Signaling Assay. The data obtained confirms that PI3K/Akt and MAPK signaling pathways mediate the effect of DDHD1 on cancer cell proliferation and survival.

### Differentially expressed proteins quantified using SWATH-MS analysis

To determinate the molecular effects induced by *DDHD1* silencing, protein profiles of Ctrl-SW480 and shDDHD1-SW480 were compared using SWATH-MS. The targeted identification of peptides in SWATH-MS datasets requires a prior generation of a spectral library that includes essential coordinates for each targeted peptide (precursor ion masses, fragment ion masses, fragment ion intensities and retention times) [15]. The peptide mixture of Ctrl-SW480 vs shDDHD1-SW480 was run twice using a Data-Dependent Acquisition (DDA) method to generate the spectral reference library (see Additional file 1: Supplementary Materials and Methods). The integrated DDA data sets were searched comparing the *Homo sapiens* UniProt fasta database using ProteinPilot 4.5 at a 1% critical False Discovery Rate (FDR) for both protein and peptide levels, allowing the identification of 2065 proteins (Additional file 3: Table S1, sheet “*Spectral reference library*”). About 60% of the proteins were identified based on at least three peptides. The resulting spectral reference library was used to develop the SWATH-MS strategy that allowed us to obtain quantitative information for 1469 proteins (Additional file 3: Table S1, sheet “*SWATH-MS data*”).

**Fig. 3:**
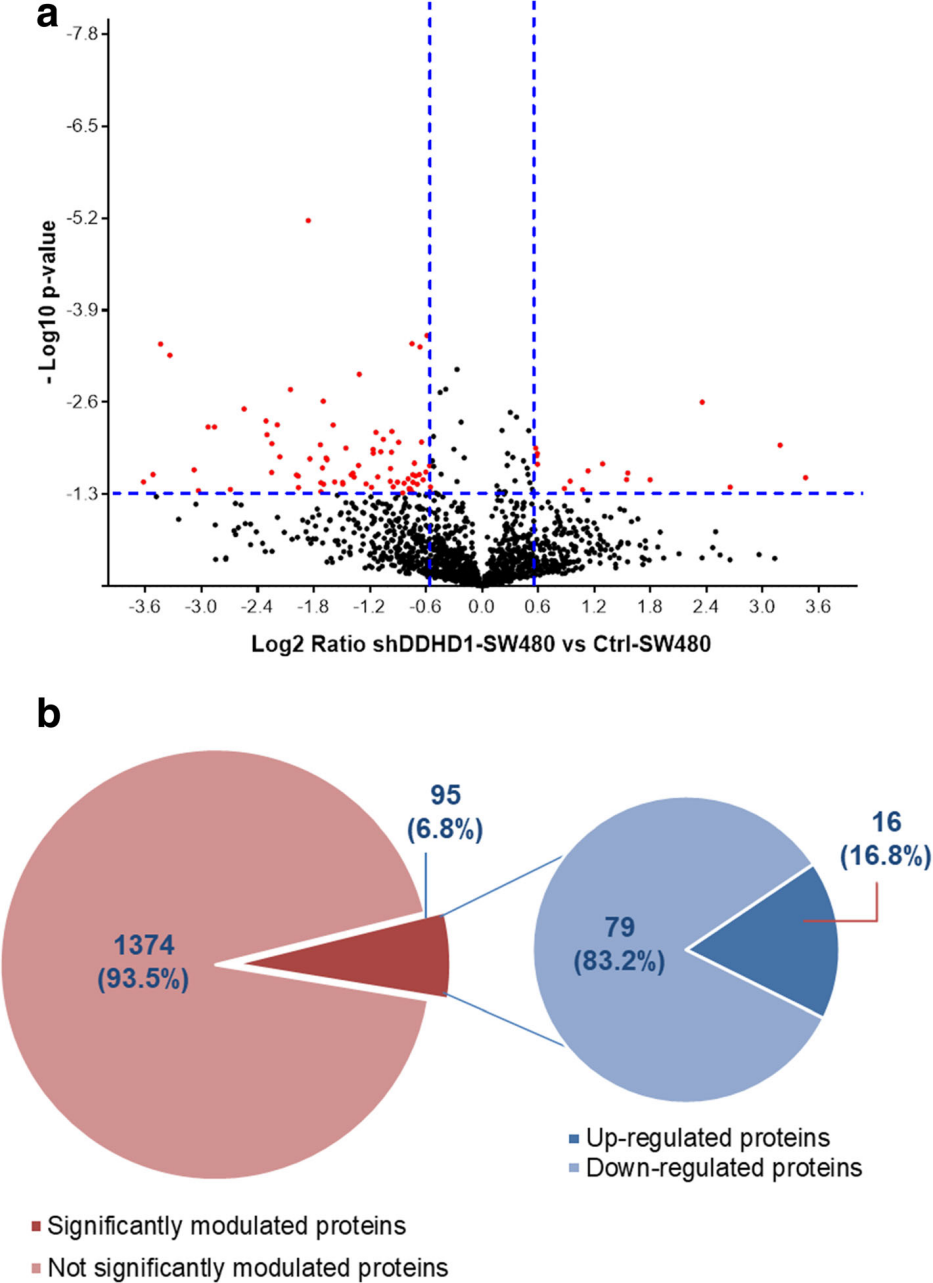
Quantitative proteomic analysis between Ctrl-SW480 and shDDHD1-SW480 cells. **a** Volcano plot of 1469 quantified proteins illustrating expression increases and decreases following DDHD1 silencing. Red dots correspond to proteins showing a fold change (FC) ≥ ±1.5 in relative abundance and a *p*-value < 0.05 (values indicated by vertical and horizontal dashed lines) and considered significantly differentially modulated in Ctrl-SW480 and shDDHD1-SW480 cells. **b** Diagram on the left illustrates the number of proteins quantified by SWATH-MS that resulted unchanged or significantly changed (Fold Change ≥ ±1.5; *p* < 0.05). Diagram on the right indicates the number of proteins up- and down-modulated by DDHD1-silencing. Numbers in parentheses indicate the percentage of total proteins

Proteins showing a fold change (FC) ≥ ±1.5 in relative abundance and a *p*-value ≤0.05 were considered differentially modulated following the *DDHD1* silencing (Fig. 3a). Among the 1469 quantified proteins, we found that the less than 7% was significantly modulated by *DDHD1* silencing, and among them more than 80% were downregulated. (Fig. 3b). In particular, we found 95 proteins significantly differentially modulated: 16 upregulated (Additional file 3: Table S1, sheet “*shDDHD1-UpRegProteins*”) and 79 downregulated (Additional file 3: Table S1, sheet “*shDDHD1-DownRegProteins*”).

**Fig. 4:**
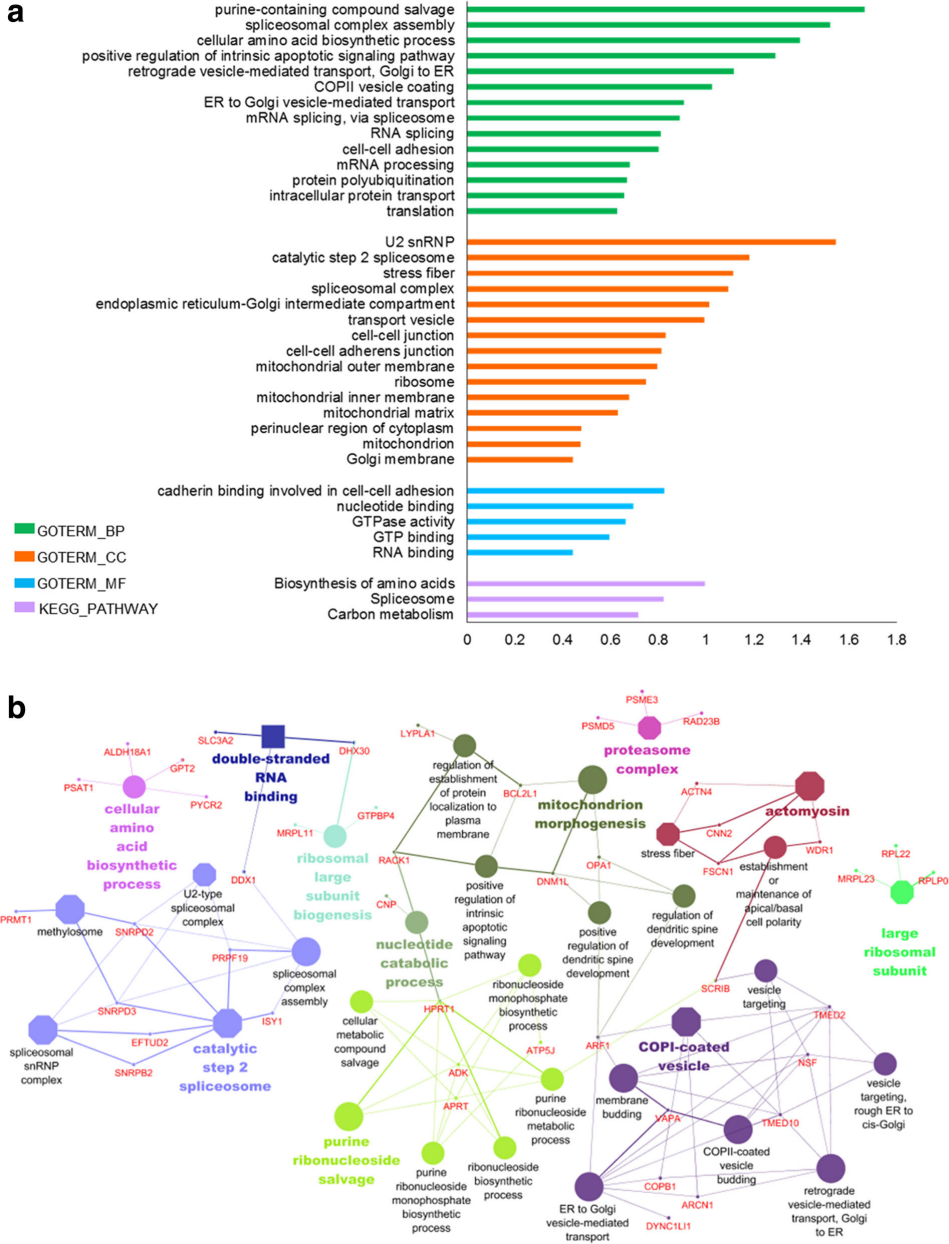
GO and KEGG pathway enrichment of shDDHD1-DownRegProteins according to DAVID functional annotation and ClueGO/CluePedia plugin. **a** The histogram shows for each GO term, Cellular Component (CC), Biological Process (BP) and Molecular Function (MF) (Fold enrichment ≥2.5, *p* ≤ 0.05) of shDDHD1-down-regulated proteins quantified using the SWATH-MS approach. **b** Network view of shDDHD1-down-regulated proteins. Terms (each represented as node) are functionally grouped based on shared proteins (kappa score ≥ 0.4) and are shown with different colors. The specific players (proteins) of each node are highlighted with the respective gene name. Node color represents the class they belong to. The size of the nodes indicates the degree of significance. Within each class, the most significant term (indicated with colored and bold characters) defines the name of the group. Octagon: CC; Ellipses: BP; Square: MF

Since it was clear the most relevant effect of *DDHD1* silencing was to negatively modulate protein expression, we focused our attention on the set of downregulated proteins, by performing the Gene Ontology (GO) and pathway enrichment analysis using the DAVID Gene Functional Classification Tool (http://david.abcc.ncifcrf.Gov), which allows to extract the biological meaning from gene/protein lists. The histogram in Fig. 4a shows the most significantly enriched categories (Fold Enrichment ≥2.5; *p*Value ≤10 − 5) for each of the three GO terms, Cellular Compartment (CC), Molecular Function (MF) and Biological Process (BP), and for KEGG pathways. Extended data for enrichment analysis is provided in Additional file 3: Table S1, sheet “*shDDHD1-DownRegProteins_DAVID*”). We found that most of enriched categories were attributable to the following seven general pathways/systems: mRNA processing/splicing, translation/ribosomes, protein ubiquitination/degradation, mitochondrial structure and activity, mechanisms of intracellular vesicles transport specifically related to RER-Golgi apparatus, biosynthesis of amino acids, purine salvage pathway.

To provide a clear explanation of the biological relevance of negative effects induced by *DDHD1* silencing, we implemented functional enrichment analysis using ClueGO+CluePedia, a Cytoscape plugin, which facilitates the visualization of functionally related genes/proteins by displaying them as a clustered network [13]. The statistical test used for the enrichment analysis was based on right-sided hypergeometric option with a Benjamini-Hochberg correction, kappa score of 0.4 and a *p*-value < 0.05 was used as the cut-off criterion. This analysis, providing (Additional file 3: Table S1, sheet “shDDHD1-DownRegProteins_ClueGO Results”) a network view for enriched GO terms, allowed us to conclude that most of enriched categories were connected by the shared proteins (Fig. 4b).

### DDHD1 overexpression supports in vitro and in vivo cancer cell growth via ERK1/2 signaling stimulation

**Fig. 5:**
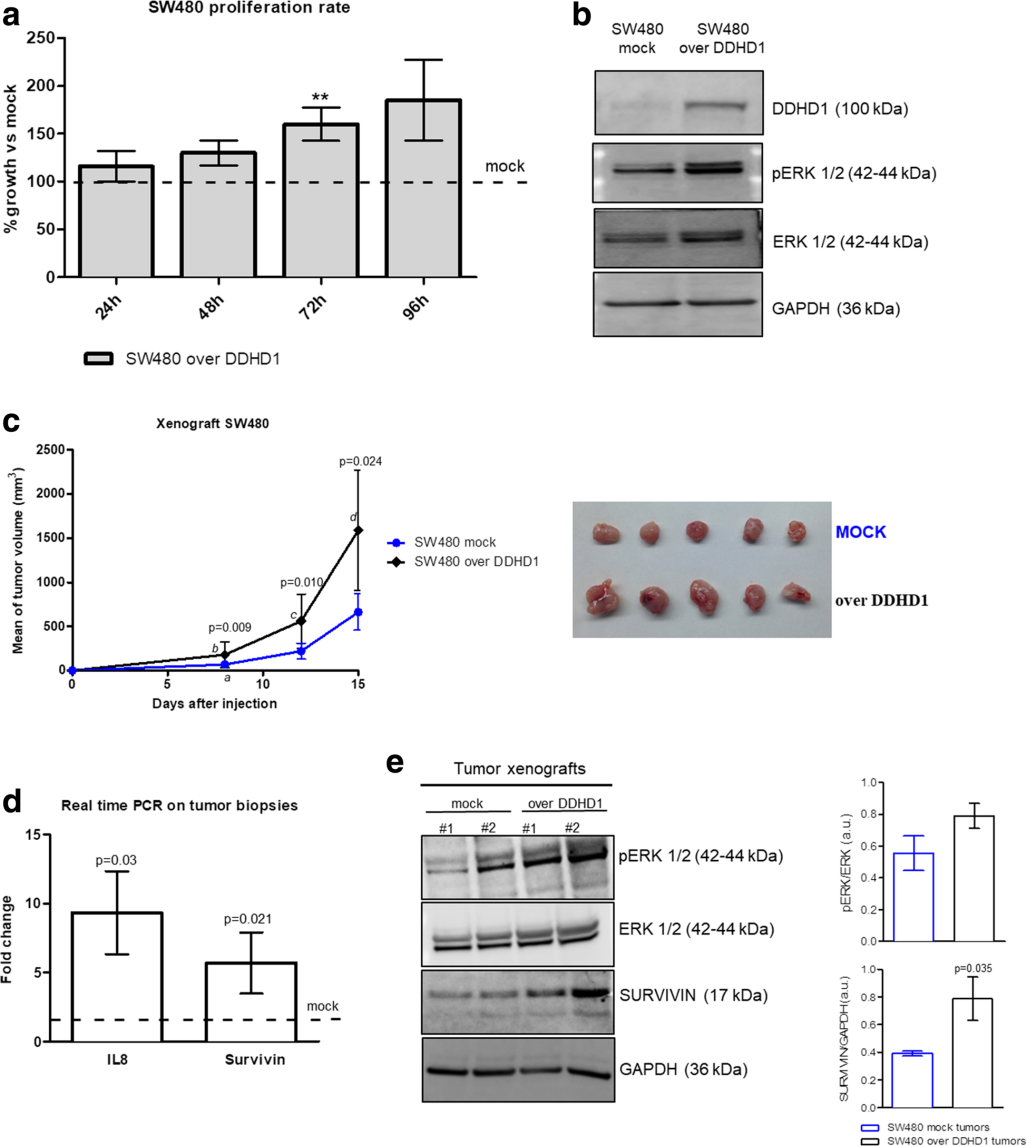
In vitro and in vivo effects of DDHD1 overexpression on colon cancer cell growth. **a** Cell viability was measured by MTT assay on SW480 cell lines after 24, 48, 72 or 96 h of transfection with mock or DDHD1 plasmid DNA. The values were plotted as % of growth vs Ctrl (cells transfected with mock plasmid). Each point represents the mean ± SD of three independent experiments. Asterisks indicate statistically significant values in comparison to control (mock) (**p ≤ 0.01). **b** Levels of DDHD1, ERK 1/2, phospho-ERK 1/2 were determined in the cell lysate of SW480 cells transfected with mock or DDHD1 plasmid DNA. Blots were probed with an antibody against GAPDH to ensure equal loading. **c** SW480 cells, transfected with mock or DDHD1 plasmid DNA were injected subcutaneously in nude mice as described. Comparison of the mean tumor volume was an index of the pro-tumor effect of DDHD1. A significant interaction was found between (group) and within (experimental time)-subject factors for volumetric data (F = 6.92, *p* = 0.028). Significant differences in terms of tumor volume were observed between the two groups (8 days *p* = 0.009; 12 days *p* = 0.010; 15 days *p* = 0.024) and within the experimental time (mock at 8 days versus starting point ^*a*,^*p* = 0.025; over DDHD1 at 8 days versus starting point ^*b*,^*p* = 0.0001; over DDHD1 at 12 days versus 8 days ^*c*,^*p* = 0.003; over DDHD1 at 15 days versus 12 days ^*d*,^*p* = 0.002). **d** IL8 and Survivin mRNA levels were evaluated in tumor biopsies by Real-time PCR. **e** Protein levels of ERK 1/2, phospho-ERK 1/2 and SURVIVIN were analyzed in the mice tumor biopsies

To attribute to DDHD1 a direct role in sustaining cancer cell proliferation, DDHD1 overexpression was induced in SW480 cells. The cell growth was analyzed using the MTT assay, at different time points (from 24 to 96 h) after transfection of SW480 cells with a vector containing the *DDHD1* DNA sequence. As shown in Fig. 5a, the proliferation rate of DDHD1 overexpressing cells is higher when compared to mock transfected cells (negative control), reaching a 50% increase in cell growth after 72 h of transfection. To evaluate if DDHD1 effects on cell growth was mediated by MAPK/ERK signaling, we also measured the phosphorylation rate of ERK1/2 in DDHD1 overexpressed SW480 cells compared to mock cells using Western blotting analysis. As shown in Fig. 5b, the overexpression of DDHD1 increased the levels of ERK1/2 phosphorylation, which confirms our previous hypothesis. In addition, since the enzymatic activity of DDHD1 is correlated to the release of lysophospholipid mediators [11], known to be involved in the proliferation of cancer cells, we used the conditioned medium from DDHD1-expressing cells to treat DDHD1- silenced cells. As reported in Additional file 4: Figure S2, we observed a cell growth recovery when DDHD1- silenced cells are maintained in the conditioned medium of DDHD1-expressing cells.

We further analyzed if DDHD1 overexpression may influence in vivo cell growth by using a xenograft mouse models. SW480 cells, previously transfected with mock or DDHD1 plasmid DNA, were subcutaneously inoculated into immunodeficient athymic nude mice. Xenograft tumors were measured and mice were weighed twice a week for 2 weeks, after which the mice were euthanized and the tumors were resected. We observed an increased rate of cell growth after DDHD1 overexpression. As shown in Fig. 5c (left and right panel), the tumor volume measured from SW480 overexpressed DDHD1 xenografts was significantly higher compared to controls. Gene and protein expression analyses on tumor biopsies show an increase of the pro-angiogenic cytokine IL8 and of the anti-apoptotic gene Survivin (Fig. 5d and e), confirming the increased trend of ERK1/2 phosphorylation after DDHD1 overexpression (Fig. 5e).

## Discussion

The intracellular DDHD1 is a phosphatidic acid (PA)-preferring PLA1 (PA-PLA1) [4] involved in the synthesis of lysophospholipid mediators such as LPI, which has been extensively studied for its implication in cancer development. Interestingly, in our previous study we found that the anti-cancer effects of *Citrus*-limon nanovesicles on colon cancer cells [2] was related to the downregulation of proteins involved in lipid metabolism, including DDHD1 [3].

In this study we were able to prove for the first time that DDHD1 supports colon cancer cell proliferation and survival. We found that the overexpression of DDHD1 in human colon cancer cells enhances in vitro and in vivo tumor growth, while its downregulation reduces in vitro colon cancer cell viability and increases apoptosis rate. In addition, we demonstrate that DDHD1 acts on tumor cells by modulating PI3K/Akt and ERK signaling pathways. These results are in line with previous observations that support the pro-tumor effects of DDHD1 lysophospholipid mediators, such as LPI. In neoplastic cells LPI acts by interacting with the GPR-55 and induces ERK and Akt signaling [8].

Overall, the results of our study, together with the observation that DDHD1 silencing doesn’t affect normal cells, allow us to suggest DDHD1 as a possible target for the development of anti-tumor therapies.

In order to better define the biological role of DDHD1 in tumor cells, we performed a quantitative proteomic analysis on *DDHD1* silenced cells. Interestingly, the pathway/GO term analysis revealed that most of the functional categories negatively modulated by *DDHD1* silencing were specifically related to a cancer phenotype. Among these categories, which in our study are associated to DDHD1 activity for the first time, it is noteworthy to mention the ones related to the ubiquitin/proteasome system, the amino acid biosynthetic process, the endoplasmic reticulum-Golgi network and the pathways related to the splicing mechanisms.

Our proteomic data showed that *DDHD1* silencing induced the simultaneous downregulation of pyrroline-5-carboxylate reductase 2 (PYCR2), aldehyde dehydrogenase 18 family member A1 (ALDH18A1), and phosphoserine aminotransferase 1 (PSAT1), three enzymes involved in the arginine-proline metabolism, which is one of nonessential amino acid metabolism pathways. In recent years, numerous studies demonstrated the relationship between cancer and nonessential amino acid metabolism pathways, underlining the potential role of the related metabolic enzymes to be effective and relevant cancer therapy targets [16, 17].

Another functional category downregulated following *DDHD1* silencing, is the one related to endoplasmic reticulum (ER)-Golgi system, known for its implication in regulating cancer cell behavior; the correct balance between forward and backward transport is essential to post-translationally modify the immature proteins [18]. It is known that, compared to normal cells, the ER-Golgi trafficking is increased in cancer cells [18] where it was correlated with increased proliferation and migration ability [19]. Moreover, it was demonstrated that the inhibition of intracellular protein trafficking induces anti-cancer effects [20], thus proteins involved in ER to Golgi vesicle-mediated transport are indicated as promising targets for developing new therapeutic strategies. Our proteomic analysis confirmed that DDHD1 downregulation elicited the inhibition of several proteins involved in ER-Golgi activity, such as VAPA, TMED2, COPB1, ARCN1, TMED10 and NSF. This could be one of the mechanisms responsible for cell proliferation and survival inhibition that was observed in *DDHD1* silenced cells. Furthermore, it is noteworthy that even if DDHD1 is specifically described as a cytosolic protein, other two members of the intracellular phospholipase A1 family, p125/Sec23ip and KIAA0725p/DDHD2, all having a DDHD domain, are described as more stably associated with the Golgi/endoplasmic reticulum (ER)-Golgi intermediate compartment (ERGIC) and ER exit sites, respectively [21]. In addition, in a previous study by Kunduri, the authors demonstrated that proteins belonging to the PA-PLA1 family are necessary to enable the transit of N-linked glycosylated proteins from the ER to the Golgi complex [22]. For the first time our data also indicates a possible role of DDHD1in ER-Golgi targeting.

Although it was stimulating for us to find a connection of DDHD1 activity to cellular pathways that were not previously described, it was equally important to have evidence of the effects induced by *DDHD1* silencing on the systems already known to be DDHD1-related, such as mitochondria. As previously discussed, DDHD1 is a phosphatidic acid (PA)-preferring phospholipase that was also described as a regulator of mitochondrial dynamics. Specifically, Baba et al. noted that PA-PLA1/DDHD1 is able to promote mitochondrial fission, a biological process recently recognized as pathogenic in various cancer models and correlated to different stages of tumor progression [23]. Among the 79 proteins downregulated in *DDHD1* silenced cells, we found 17 mitochondrial proteins (both with structural and enzymatic function), including Dynamin related protein 1 (DNM1L) and Dynamin-like 120 kDa protein (OPA1), both involved in regulation of the balance between mitochondrial fusion and mitochondrial fission [23, 24]. Studies on lung and hepatocellular carcinoma provided evidence showing that the induction of mitochondrial injury, due to the inhibition of Drp1, correlates with a decrease of the proliferation rate and an increase of apoptosis [25, 26].

## Conclusions

In conclusion, this study provides the first evidence confirming the role of DDHD1 in cancer, providing the possibility to define a new target to design more effective therapies for colon cancer patients. In addition, the proteomic analysis provided us with new knowledge on DDHD1 cytoplasmic activity, highlighting its involvement in both known and previously unrecognized intracellular pathways and identifying multiple mechanisms that may explain the suppressed cancer cell growth induced by *DDHD1* silencing. These findings are still preliminary and important questions need to be addressed. Therefore, further studies will be necessary to better understand and outline DDHD1 enzymatic activity in cancer, as well as its interaction with other proteins.

## Additional files

Additional file 1: Supplementary Material and Methods. (DOCX 24 kb)

Additional file 2: **Figure S1.** DDHD1 silencing. To evaluate DDHD1 silencing **a**. Real-time PCR and **b**. Western blot analysis were performed on SW480, HCT116, HS5 and HUVEC transfected for 48 or 72 h with scrambled siRNA or DDHD1 siRNA. (TIFF 6629 kb)

Additional file 3: **Table S1.** Data from SWATH-MS Gene Ontology analysis. (XLSX 740 kb)

Additional file 4: **Figure S2.** Effects of DDHD1-expressing cells conditioned medium on DDHD1-silenced cell growth. Cell viability was measured by MTT assay on *DDHD1*-silenced SW480 cells in the presence of the conditioned medium (CM) of mock cells and *DDHD1* overexpressing cells. (TIFF 3275 kb)
