# Supplementary material for: The phospholipase DDHD1 as a new target in colorectal cancer therapy
Source: J Exp Clin Cancer Res. 2018 Apr 13;37:82. doi: 10.1186/s13046-018-0753-z (PMC5899352; doi:10.1186/s13046-018-0753-z)
Supplement: Supplementary file 1 — Supplementary Material and Methods. (DOCX 24 kb) [file 13046_2018_753_MOESM1_ESM.docx]

**Supplementary Material and Methods**

**RNA extraction and Real-Time PCR**

SW480, HCT116, HS5 and HUVEC cells were seeded in 24-well plate, transfected with 10 nM of scrambled or *DDHD1* siRNA and grown for 48 and 72 hours. Tumor biopsies, soon after removal, were stored in RNAlater solution (Applied Biosystems, Foster City, California, USA). Each sample was lysed in a tissue homogenizer. RNA was purified using the commercially available Illustra RNAspin Mini Isolation Kit (GE Healthcare, Little Chalfont, Buckinghamshire, UK), according to manufacturer’s instructions. Total RNA was reverse-transcribed to cDNA using the High Capacity cDNA Reverse Transcription Kit (Thermo Fisher Scientific). RT-QPCR was performed in 48-well plates using the Step-One Real-Time PCR system (Applied Biosystem). For quantitative SYBR®Green realtime PCR, the following primers were used: *DDHD1* (5’ TTTCTCAACCCAGCTAAAGAACCTA 3’, 5’ TGATCCAACTCCAATGCAGAAT 3’), *SURVIVIN* (5’CTCAAGGACCACCGCATCTC’3, 5’CAGCCTTCCAGCTCCTTGAA’3), *IL8* (5’GAATGGGTTTGCTAGAATGTGATA’3, 5’CAGACTAGGGTTGCCAGATTTAAC’3) and *GAPDH* (5’ CAATGACCCCTTCATTGACC 3’, 5’ TTGATTTTGGAGGGATCTCG 3’), all obtained from Invitrogen (Foster City, CA, USA). Real-time PCR was performed in triplicates for each data point. Relative changes in gene expression between control and treated samples were determined with the ΔΔCt method. Levels of the target transcript were normalized to *GAPDH* endogenous control, constantly expressed in all samples (ΔCt). For ΔΔCt values, additional subtractions were made between treated samples and control ΔCt values. Final values were expressed as fold of induction.

**Western blotting**

SW480 and HCT116 cells were seeded in a 6-well plate, transfected with 10 nM of scrambled or DDHD1 siRNA and grown for 48 or 72 hours. Similarly, SW480 cells transfected with 2,5 μg of mock or DDHD1 plasmid DNA were seeded in a 6-well plate and grown for 72 hours.

Total protein cell lysates were obtained from cells and tumor xenografts and analyzed by SDS-PAGE followed by Western blotting. Antibodies used in the experiments were: anti-GAPDH, anti- SURVIVIN (Santa Cruz Biotechnology, Santa Cruz, CA, USA), anti-DDHD1 (Novus), anti-ERK 1/2, anti-phospho-ERK 1/2 (Cell Signaling Technology, MA, USA).

**Viability assay (MTT assay)**

Cell viability was assessed with Methyl-thiazol-tetrazolium (MTT) assay as previously described [[16](#_ENREF_16)]. Briefly, SW480, HCT116, HS5 and HUVEC cells were seeded in a 24-well plate, transfected with 10 nM of scrambled or *DDHD1* siRNA and grown for 48 and 72 hours. Similarly, SW480 cells, previously transfected with 2,5 μg of mock or DDHD1 plasmid DNA, were seeded and grown for 24, 48, 72 and 96 hours. The absorbance was measured at 540 nm.

**Annexin V assay**

To detect tumor cell apoptosis, an Annexin V-fluorescein isothiocyanate (FITC) assay was used. Specifically, SW480, HCT116, HS5 and HUVEC cells were seeded in a 6-well plates, transfected with scrambled or DDHD1 siRNA and cultured for 72 hours. After incubation, cells were suspended in Annexin V binding buffer and mixed with 5μl of Annexin V-FITC (BD-Biosciences, San Jose, CA). The cells were then incubated at room temperature in the dark for 15 min. Samples were subjected to flow cytometry analysis to detect cell apoptosis levels. Cells positive for Annexin V-FITC, were considered to represent apoptotic cells. Stained cells were acquired on FACS Calibur (BD Biosciences San Jose, CA) and analysed using FlowJo software (Tree Star, Ashland OR).

**Proteomic analysis**

*Protein extraction and digestion*

All chemicals used for protein extraction and digestion were of analytical grade, and Milli-Q water was employed in all buffers and solutions. SW480 cells transiently transfected with scrambled (Ctrl-SW480) or DDHD1 siRNA (shDDHD1-SW480) were dissolved in 100 μL of 50% tetrafluoroethylene (Sigma-Aldrich) in PBS, vigorously vortexed and sonicated for 7 min in an ice bath and finally incubated with constant shaking for 2 h at 60 °C. Proteins were reduced with 5 mM dithiothreitol (Sigma Aldrich SRL, Milan, Italy) for 30 min at 60 °C and alkylated with 25mM iodoacetamide (Sigma-Aldrich) for 30 min in the dark at room temperature. Before adding mass spectrometry-grade trypsin (Pierce™ Trypsin Protease, Fisher Scientific Italia, Rodano-Milan, Italy), the samples were diluted 5-fold with 100 mM ammonium bicarbonate pH 8.0. Protein samples were digested by adding trypsin at a ratio of 1:50 (w/w) for 18 h at 37 °C with constant shaking, in presence of 2 mM CaCl2. To stop digestion 50 μL of 2.5% trifluoroacetic acid (TFA HPLC Grade, Sigma-Aldrich) were added to the samples. Digested samples were then centrifuged at 14,000g for 10 min at 4 °C. The resulting supernatant, containing the peptide mixture, was extracted using the 100 μL Bond Elute OMIX C18 pipette tips (Agilent Technologies Italia SpA, Cernusco sul Naviglio – Milan, Italy). Eluted peptide mixtures were vacuum dried and reconstituted in 5% acetonitrile 0.1% formic acid for mass spectrometry analyses. Three biological replicates of each sample were used for the following proteomic analysis.

*Generation of the reference spectral library*

Approximately 2 μg of tryptic peptides of each biological replicates were mixed and run twice for Data-Dependent Acquisition (DDA) analysis. The resulting list of protein/peptides was used for construction of the Sequential Window Acquisition of all THeoretical (SWATH) reference spectral library. The sample was analyzed via reverse-phase high-pressure liquid chromatography electrospray ionization tandem mass spectrometry (RP-HPLC-ESI-MS/MS) using a TripleTOF® 5600 mass spectrometer (AB SCIEX, Milan, Italy). The mass spectrometer was coupled to a nanoLC Eksigent 425 system (AB SCIEX). RP-HPLC was performed with a trap and elution configuration using an Acclaim™ PepMap™ 100 Nano Trap Column 100 μm x 2 cm, C18, 5 μm, 100 Å (Fisher Scientific Italia) and an Acclaim™ PepMap™ RSLC Nano Column 75 μm x 250 mm, C18, 2 μm, 100 Å (Fisher Scientific Italia). The reverse-phase LC solvents were: solvent A (0.1% formic acid in water) and solvent B (2% water and 0.1% formic acid in acetonitrile). The sample was loaded in the trap column at a flow rate of 5 μL/min for 10 min using a solvent, from loading pump, containing 2% acetonitrile and 0.1% v/v TFA in water and eluted at a flow rate of 300 nL/min using a gradient method according to which solvent B is linearly increased from 2% to 10% within 10 min, from 10% to 30% within 110 min and then to 60% within 15 min; afterwards, phase B is further increased to 95% within 2 min. Phase B is maintained at 95% for 10 min to rinse the column. Finally, B is lowered to 2% over 2 min and the column re-equilibrated for 21 min (170 min total run time). The eluting peptides were on-line sprayed in the Triple TOF 5600 Plus mass spectrometer, that it is controlled by Analyst TF 1.7 software (AB SCIEX).

The mixed sample used to generate the SWATH-MS spectral library was subjected to two DDA runs. The mass range for MS scan was set to m/z 400– 1250 and the MS/MS scan mass range was set to m/z 230–1,500. Using the mass spectrometer, a 0.25 s survey scan (MS) was performed, and the top 50 ions were selected for subsequent MS/MS experiments employing an accumulation time of 0.065 s per MS/MS experiment for a total cycle time of 3.5485 s. Precursor ions were selected in high resolution mode (>30,000), tandem mass spectra were recorded in high sensitivity mode (resolution >15,000). The selection criteria for parent ions included an intensity of greater than 500 cps and a charge state ranging from + 2 to + 5. A 15 s dynamic exclusion was used. The ions were fragmented in the collision cell using rolling collision energy, and a collision energy spread (CES) of 5 V.

The two DDA MS raw files were combined and subjected to database searches in unison using ProteinPilot™ 4.5 software (AB SCIEX) with the Paragon algorithm by using the following parameters: iodoacetamide cysteine alkylation, digestion by trypsin and no special factors. The search was conducted through identification efforts in a uniprot-allhumanProteome2017 dataset (http://www.uniprot.org/, downloaded in July 2017, with 71591 protein sequence entries) containing whole Homo sapiens proteins. A false discovery rate analysis was also performed.

*SWATH-MS analysis and targeted data extraction*

Three biological of each sample were subjected to the cyclic data independent acquisition (DIA) of mass spectra. Data were acquired by repeatedly cycling through 40 consecutive 15- Da precursor isolation windows (swaths). For these experiments, the mass spectrometer was operated using a 0.1 s survey scan (MS). The subsequent MS/MS experiments were performed across the mass range of 100 to 1600 m/z on all precursors in a cyclic manner using an accumulation time of 0.03 s per SWATH window for a total cycle time of 1.3490 s. Ions were fragmented for each MS/MS experiment in the collision cell using rolling collision energy, and CES was set to 15. The spectral alignment and targeted data extraction of DIA samples were performed using PeakView v.2.2 (AB SCIEX) with the reference spectral library. All eight DIA files were loaded and exported in .txt format in unison using an extraction window of 15 min and the following parameters: three hundred peptides/protein, seven transitions/peptide, peptide confidence level of 90%, excluded shared and modifies peptides, and an extracting ion current (XIC) width of 75 ppm. This export procedure generated three distinct files containing the quantitative output for (1) the peak area under the intensity curve for individual ions, (2) the summed intensity of individual ions for a given peptide, and (3) the summed intensity of peptides for a given protein. For each protein, seven individual ion intensities were summed as peptide intensity, until three hundred peptides intensities were summed as protein intensity. The protein list with False Discovery Rate (FDR) lower than 5% generated by analyzing SWATH data with PeakView 2.2, was exported to MarkerView 1.2.1 (AB SCIEX) for statistical analysis.

*Bioinfomatic analysis*

The Gene Ontology and KEGG pathway analysis of proteins down regulated by DDHD1-silencing (shDDHD1-DownRegProteins) was initially performed using the online tool DAVID (http://david.abcc.ncifcrf.gov/). The ClueGO v2.3.3+CluePedia v1.3.3, a Cytoscape v3.4.0 plug-in was used to visualize GO terms and pathways in functionally organized networks reflecting the relations between the biological terms based on the similarity of their linked gene/proteins [18]. In order to make cluster/group comparison and highlight functional differences, the set of shDDHD1-DownRegProteins was uploaded in ClueGO using the Cytoscape environment.

For the enrichment of biological terms and groups, we used the two-sided (Enrichment/Depletion) tests based on the hyper-geometric distribution. We set the statistical significance to 0.05 (p ≤ 0.05), and we used the Benjamini-Hochberg adjustment to correct the p-value for the terms and the groups created by ClueGO. These were the used parameters: kappa score threshold set to 0.4; GO tree interval: 3–8; Leading Group: Highest Significance.
