# Supplementary figures and images for: The phospholipase DDHD1 as a new target in colorectal cancer therapy
Source: J Exp Clin Cancer Res. 2018 Apr 13;37:82. doi: 10.1186/s13046-018-0753-z (PMC5899352; doi:10.1186/s13046-018-0753-z)

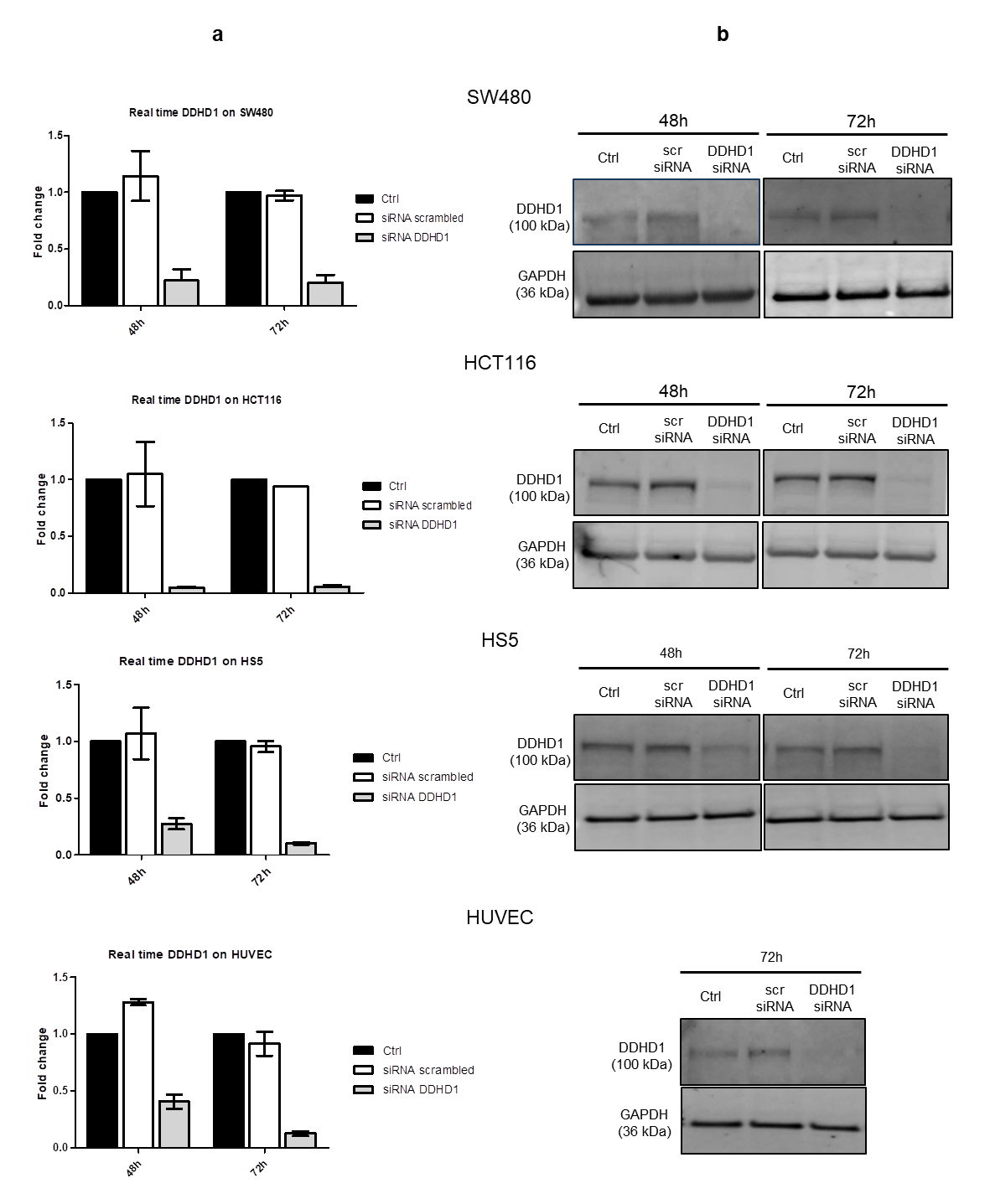

Supplement: Supplementary file 2 — Figure S1. DDHD1 silencing. To evaluate DDHD1 silencing a. Real-time PCR and b. Western blot analysis were performed on SW480, HCT116, HS5 and HUVEC transfected for 48 or 72 h with scrambled siRNA or DDHD1 siRNA. (TIFF 6629 kb) [file 13046_2018_753_MOESM2_ESM.tiff]

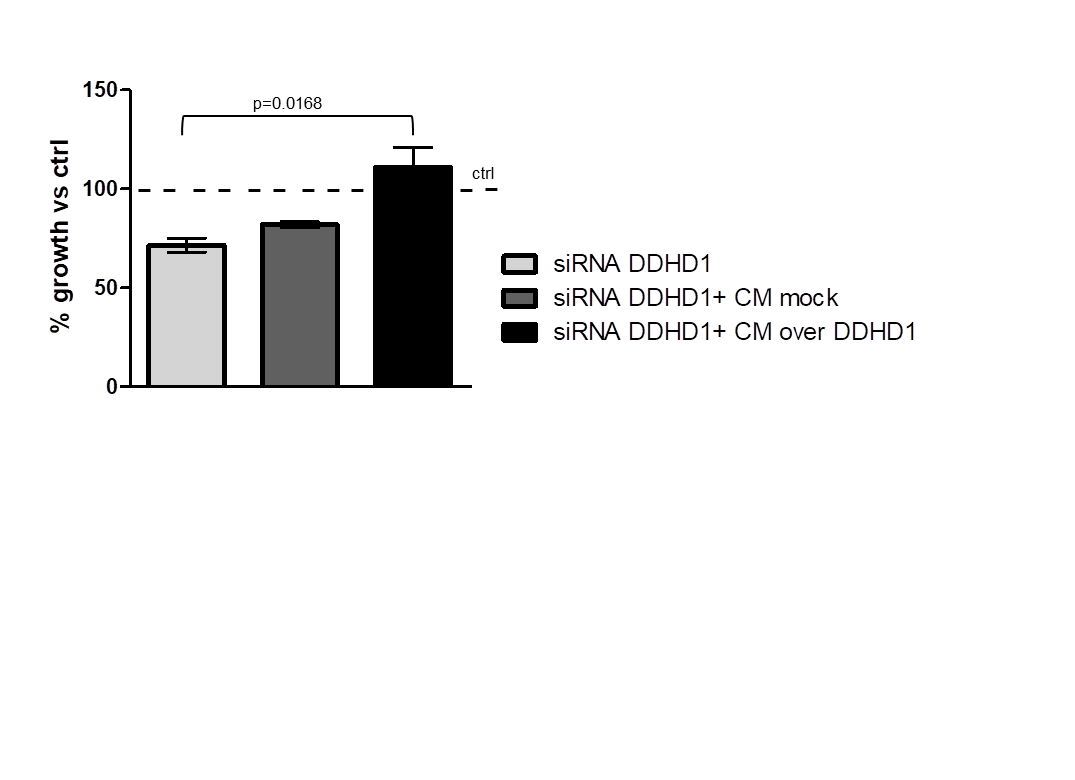

Supplement: Supplementary file 4 — Figure S2. Effects of DDHD1-expressing cells conditioned medium on DDHD1-silenced cell growth. Cell viability was measured by MTT assay on DDHD1-silenced SW480 cells in the presence of the conditioned medium (CM) of mock cells and DDHD1 overexpressing cells. (TIFF 3275 kb) [file 13046_2018_753_MOESM4_ESM.tiff]
